# The Travelling-Wave Primate System: A New Solution for Magnetic Resonance Imaging of Macaque Monkeys at 7 Tesla Ultra-High Field

**DOI:** 10.1371/journal.pone.0129371

**Published:** 2015-06-11

**Authors:** Tim Herrmann, Johannes Mallow, Markus Plaumann, Michael Luchtmann, Jörg Stadler, Judith Mylius, Michael Brosch, Johannes Bernarding

**Affiliations:** 1 Department of Biometrics and Medical Informatics, OvG University, Magdeburg, Germany; 2 Leibniz Institute for Neurobiology (LIN), Magdeburg, Germany; 3 Center for Behavioral Brain Sciences, Magdeburg, Germany; University of Minnesota, UNITED STATES

## Abstract

**Introduction:**

Neuroimaging of macaques at ultra-high field (UHF) is usually conducted by combining a volume coil for transmit (Tx) and a phased array coil for receive (Rx) tightly enclosing the monkey’s head. Good results have been achieved using vertical or horizontal magnets with implanted or near-surface coils. An alternative and less costly approach, the travelling-wave (TW) excitation concept, may offer more flexible experimental setups on human whole-body UHF magnetic resonance imaging (MRI) systems, which are now more widely available. Goal of the study was developing and validating the TW concept for in vivo primate MRI.

**Methods:**

The TW Primate System (TWPS) uses the radio frequency shield of the gradient system of a human whole-body 7 T MRI system as a waveguide to propagate a circularly polarized B1 field represented by the TE11 mode. This mode is excited by a specifically designed 2-port patch antenna. For receive, a customized neuroimaging monkey head receive-only coil was designed. Field simulation was used for development and evaluation. Signal-to-noise ratio (SNR) was compared with data acquired with a conventional monkey volume head coil consisting of a homogeneous transmit coil and a 12-element receive coil.

**Results:**

The TWPS offered good image homogeneity in the volume-of-interest Turbo spin echo images exhibited a high contrast, allowing a clear depiction of the cerebral anatomy. As a prerequisite for functional MRI, whole brain ultrafast echo planar images were successfully acquired.

**Conclusion:**

The TWPS presents a promising new approach to fMRI of macaques for research groups with access to a horizontal UHF MRI system.

## Introduction

Ultra-high field (UHF) functional magnetic resonance imaging (fMRI)[1] in monkey brain imaging imposes novel technical requirements, especially for excitation and signal detection. Good results have been shown by some research groups performing fMRI on monkeys using vertical UHF magnetics [2,3], with the advantage of lower acquisition costs and sitting monkey position but the disadvantage of limited space inside. For larger animals such as macaques, widely available horizontal human whole-body MRI systems offer more space for several experimental setups. Here, good results were presented at lower fields by Vanduffel et al. 2001 several years ago [4]. Ultra-high field MRI offers the chance to acquire images with a higher signal-to-noise ratio. For macaque brain imaging on a UHF 7 T human whole-body MRI system, a combination of volume transmit (Tx) [5] and phased array receive (Rx) only coils [6] or a multi-transmit coils (Tx/Rx) [7] is usually deployed, both tightly enclosing the macaque's head. MRI volume transmit radio frequency (RF) coils are not always suitable for fMRI on macaques because the RF coil tightly encloses their heads in order to generate a homogenous B_1_^+^ excitation, often limiting the number of necessary auditory or visual stimulation units that can be attached, which restricts some experimental setups. Furthermore, using a fixation device for macaques cuts the use of volume coils and leaves space only for multi-transmit (Tx/Rx) or phased array (Rx) coils on a UHF 7 T human whole-body MRI system. Important requirements for excitation and reception are a highly homogeneous irradiated B_1_^+^ field, which serves to achieve excitation and a high signal-to-noise ratio (SNR). In particular, many fMRI experiments require stimulation units near the head, e.g. for visual or auditory stimulation. Designing the experimental setting such that the RF coils and the monkey fixation unit leave enough space and flexibility for different experimental units would thus greatly enhance the potential for more experiments.

To achieve the most homogenous B_1_^+^ excitation, a whole-body resonator is usually the best choice. For MRI systems with lower fields (1.5 T and 3 T) this homogeneous B_1_^**+**^ field excitation is usually provided by whole-body resonators in the birdcage design [5]. At present this body resonator is not available on UHF human whole-body MRI systems because the standard birdcage architecture is of only limited use at system frequencies beyond 200 MHz. The travelling-wave (TW) [8] excitation approach makes excitation possible on human whole-body UHF MRI systems and can serve as transmitter to achieve homogeneous B_1_^+^ excitation for neuroimaging applications with crab-eating macaques [9]. This approach offers more space by using phased array coils for flexible experimental setups for close-fitting auditory or visual stimulation with fMRI and allows for usage of a monkey fixation device. In the TW excitation approach, the RF shield of the UHF whole-body MRI system acts as a waveguide to direct the excitation wave to the object of interest. With an antenna, an electromagnetic wave radiates into the magnet bore; if the bore diameter is sufficiently large, RF energy attenuation is negligibly low and the wave propagates through the bore. The frequency condition meeting the above restrictions is known as the cut-off frequency. For an RF shield diameter of, e.g. 64 cm, the cut-off frequency for the TE_11_ mode of the circular waveguide is 275 MHz, which is sufficiently smaller than the 7 T Larmor frequency of 297.2 MHz for 1H (hydrogen) [10]. This lower cut-off frequency allows waves to be propagated inside the RF shield of the human whole-body UHF MRI system. The advantage of the TW excitation approach lies in its simple implementation in a human whole-body UHF MRI system. By adapting this approach to macaque fMRI, we developed the TW Primate System and evaluated and verified it in vivo experiments.

## Materials and Methods

### Experimental setup

**Fig 1:**
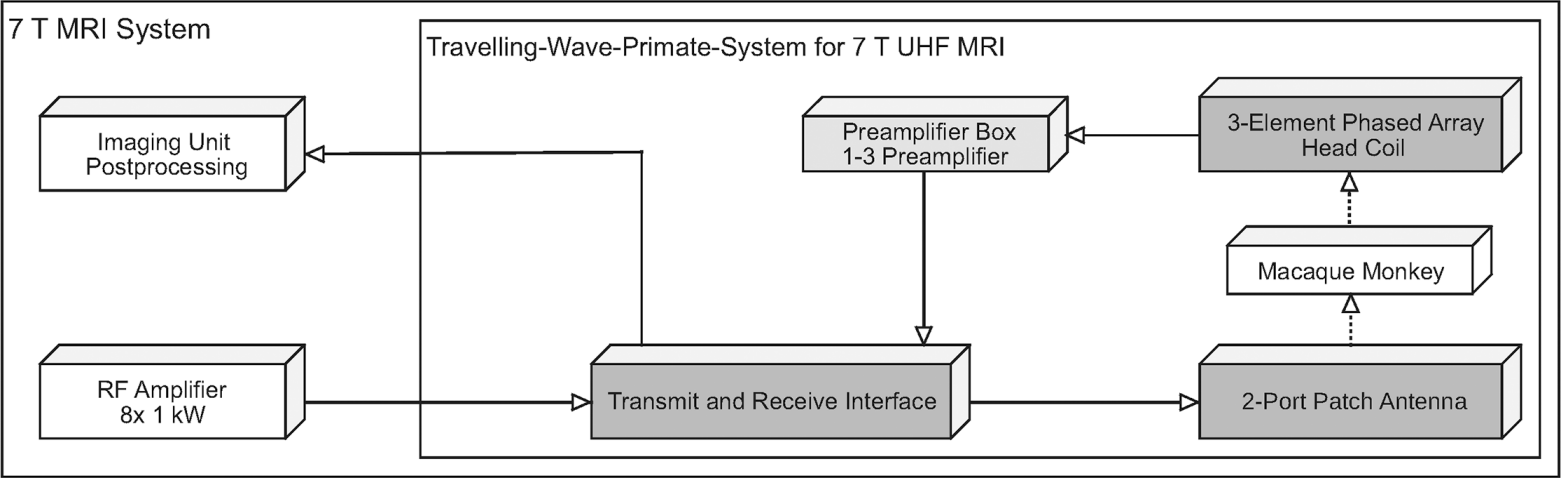
Scheme of the Travelling-Wave Primate System. Including the main parts: patch antenna to generate a circularly polarized B1 field; RF interface box for driving the patch antenna, 3-element phased array primate head coil, and an external preamplifier interface box.

**Fig 2:**
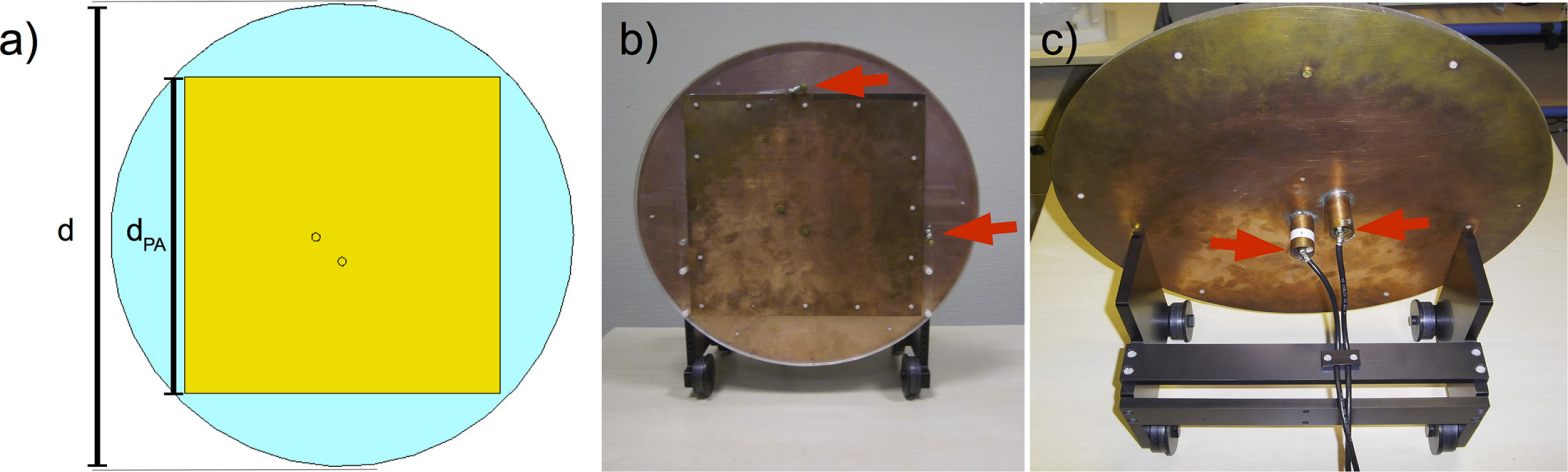
Patch antenna for transmit of the B1 field. CAD model of the patch antenna with dimensions (**a**). Front view of the constructed patch antenna with tuning capacitors (red arrows) (**b**). Back side view of the patch antenna with matching capacitors (red arrows) and transport carriage (**c**).

**Fig 3:**
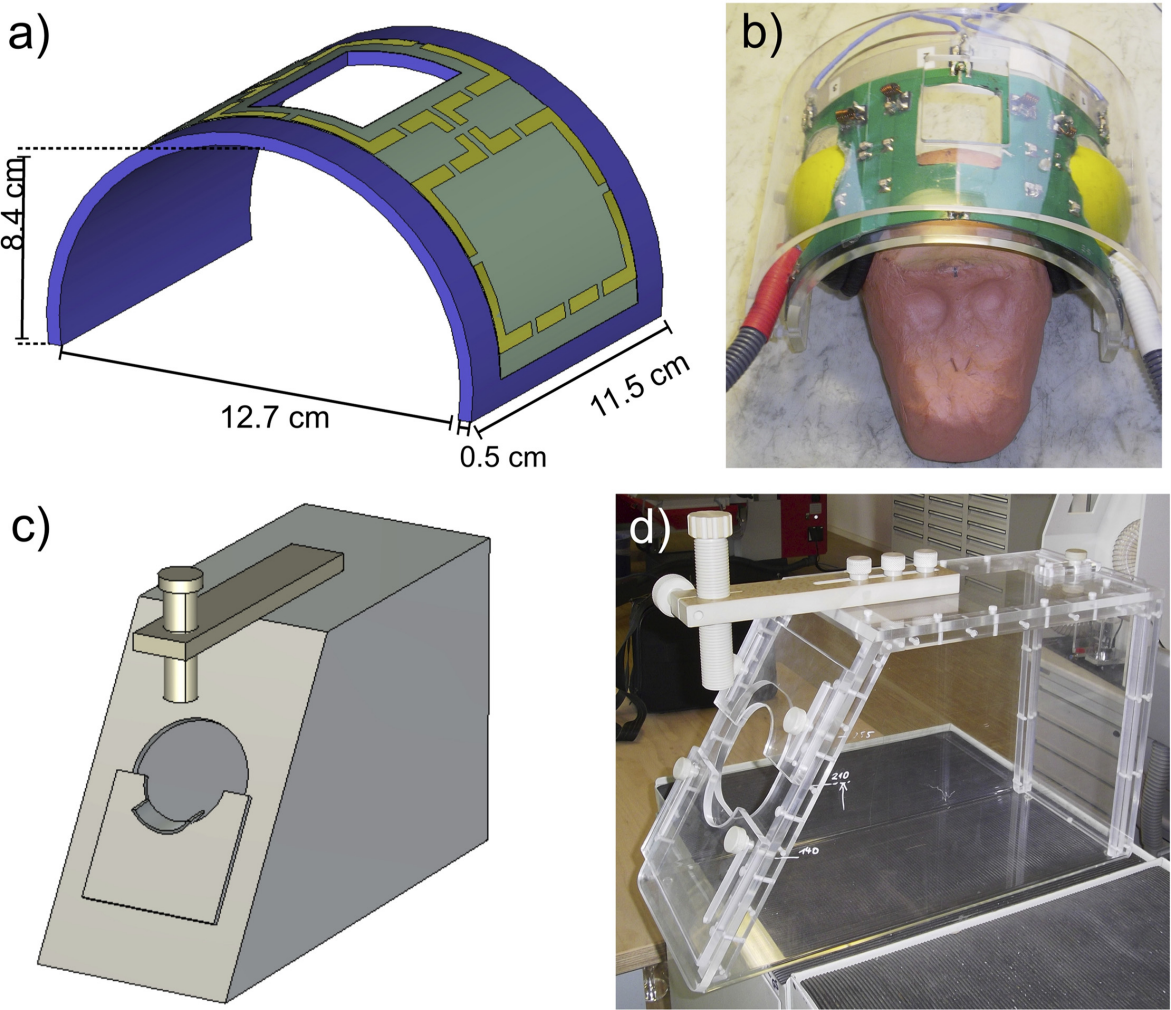
3-element phased array receive-only primate head coil and monkey fixation device. CAD Model of the 3-element phased array primate head coil with dimensions (**a**). 3-element phased array receive-only primate head coil with clay model of monkey head (**b**). CAD model of the fixation device (**c**). In-house designed fixation device with PMMA as non-magnetic base material (**d**).

The TW Primate System consists of four main components (Fig 1): a patch antenna to generate a circularly polarized B_1_ field, an RF interface box for driving the patch antenna, a 3-element phased array monkey head coil for receive-only together with an external preamplifier box, and a fixation device for the crab-eating macaques. The 2-port patch antenna (system frequency 297.2 MHz), which generates a circularly polarized B_1_ field for a more efficient B_1_^+^ field, was designed to work inside the RF shield of the human whole-body 7 T MRI system. Its antenna was designed (Fig 2A) and optimized using field simulation software CST Microwave Studio 2014 [11]. To reduce the diameter, the patch antenna was fabricated on a polymethylmethacrylate (PMMA) substrate. In this case the diameter d of the complete antenna was reduced to 44 cm, which fits optimally into the 7 T inner bore diameter of 60 cm. The diameter of the square copper patch d_PA_ was 30 cm. The 2-port patch antenna was driven with a 90° phase shift to produce the circularly polarized B_1_ field for the excitation. The nominally supplied RF power was limited to 8 kW. Trim capacitors for matching and tuning were used to optimize the RF energy balance (Fig 2B and 2C). In addition, the antenna was tuned and matched inside the 7 T MRI system. This provides a strong RF energy coupling between the patch antenna and the RF shield of the 7 T MRI system. The patch antenna was driven by an in-house designed RF interface box, which includes two Wilkinson power dividers, two transmit/receive switches, and two low-noise preamplifiers (Siemens 7576–312 modified to 7 T by the company Stark Contrast). For positioning the patch antenna, a transport carriage consisting of non-magnetic polyethylene (PE) material was designed and constructed (Fig 2C). The antenna was installed on the front side of the magnet bore 120 cm away from the iso-center. It had a Q-factor of 27 for both ports and a decoupling of -15 dB for the loaded condition. For the high-sensitivity in vivo monkey brain imaging a 3-element phased array head coil (Fig 3A) with passive detuning and a high B_1_ filling factor was designed and optimized using the field simulation software CST Microwave Studio 2014. This monkey head coil consists of three receive elements with capacitive decoupling and had a Q-factor of 29 under unloaded conditions and 10 under loaded conditions (matched to the monkey) for each element. All Q measurements were performed without preamplifier box. The decoupling between neighboring coil elements was -11 dB and -20 dB for next-nearest neighbor coil elements. Due to space limitations the 7 T low noise preamplifiers (Siemens 101-85-702 and adapter board 101-85-751) were installed in an external acrylic box. To prevent losses of the received signal by cable damping, this acrylic housing was located very close to the 3-element phased array head coil, which was prepared for acoustic functional experiments and leaves enough space for insertion of earphones (Fig 3B). The fixation device for the crab-eating macaques was designed (Fig 3C) to work inside the human whole-body 7 T MRI system and was fabricated with PMMA as a non-magnetic material (Fig 3D). This type of device is usually used for monkey imaging in horizontal MRI systems [12,13]. It places the crab-eating macaque in the so-called sphinx position [12] to reduce motion artifacts and artifacts from breathing. Constructed to fixate the head, it thereby allows for fMRI studies on awake monkeys.

However, this device does not reduce artifacts created by jaw motions that affect the B_0_ field and thus can decrease the image quality for awake monkey fMRI considerably. These artifacts can be reduced by both conditioning the animal and attaching a jaw motion sensor, as shown by Keliris et al. 2007 [14].

**Fig 4:**
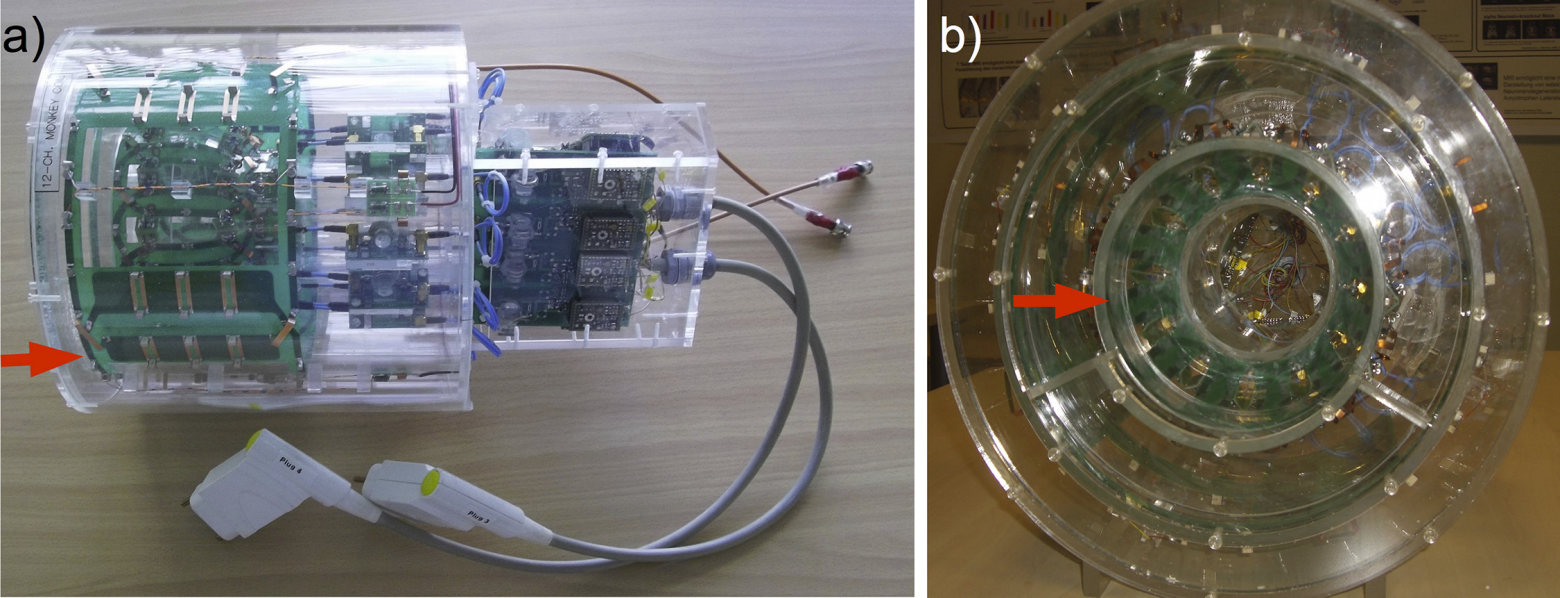
Primate volume head coil. Side view of the primate head coil with outer volume coil (Tx) (red arrow) in the Dual-Helmholtz design; side view (**a**). Primate head coil with inner receiver coil (Rx) consisting of 12 elements in phased array design (red arrow); frontal view.

To assess the performance of the TW Primate System, high-resolution anatomical datasets of the monkey brain were acquired with a conventional volume head coil for monkeys. These datasets were compared in terms of SNR and tissue homogeneity. The 7 T MRI monkey volume head coil was designed solely for the purpose of anatomical imaging and manufactured by the Neuroscience Research Institute/Gachon University in Incheon/South Korea. It (Fig 4A) consists of an outer volume coil Tx in Dual-Helmholtz (DH) design [15] and an internal receiver coil (Rx) in phased array design with 12 elements (Fig 4B). The DH excitation coil, which disposes of an active detuning circuit, has an inner diameter of 22.5 cm and a length of 13.5 cm. The housing of the DH volume coil is made of a PMMA material. This coil is equivalent to the birdcage coil architecture [5] and was also driven with the RF interface box. Disposing of a passive detuning circuit, the inner receiver coil has an inner diameter of 13.5 cm and a length of 9 cm.

### MRI system

To test the performance of the TW Primate System, all in vivo experiments were performed on a 7 T whole-body MRI system (Magnetom 7 T, Siemens Healthcare, Erlangen, Germany) equipped with a SC72d gradient coil with a maximum amplitude of 70 mT/m and maximum slew rate of 200 mT/m/ms. The coil was equipped with a 147 cm long RF shield with a diameter of 64 cm. The system control software version was VB17 (UHF). The linear pulse power amplifier (LPPA 13080W, COMET Stolberg, Stolberg, Germany) providing the RF power was equipped with eight 1 kW RF power amplifiers combined with a power combiner to generate in sum about 8 kW. By including the power losses from RF power amplifiers to the RF coil or patch antenna, the supplied RF power is estimated at approx. 6.5 kW.

### In vivo experiments

**Fig 5:**
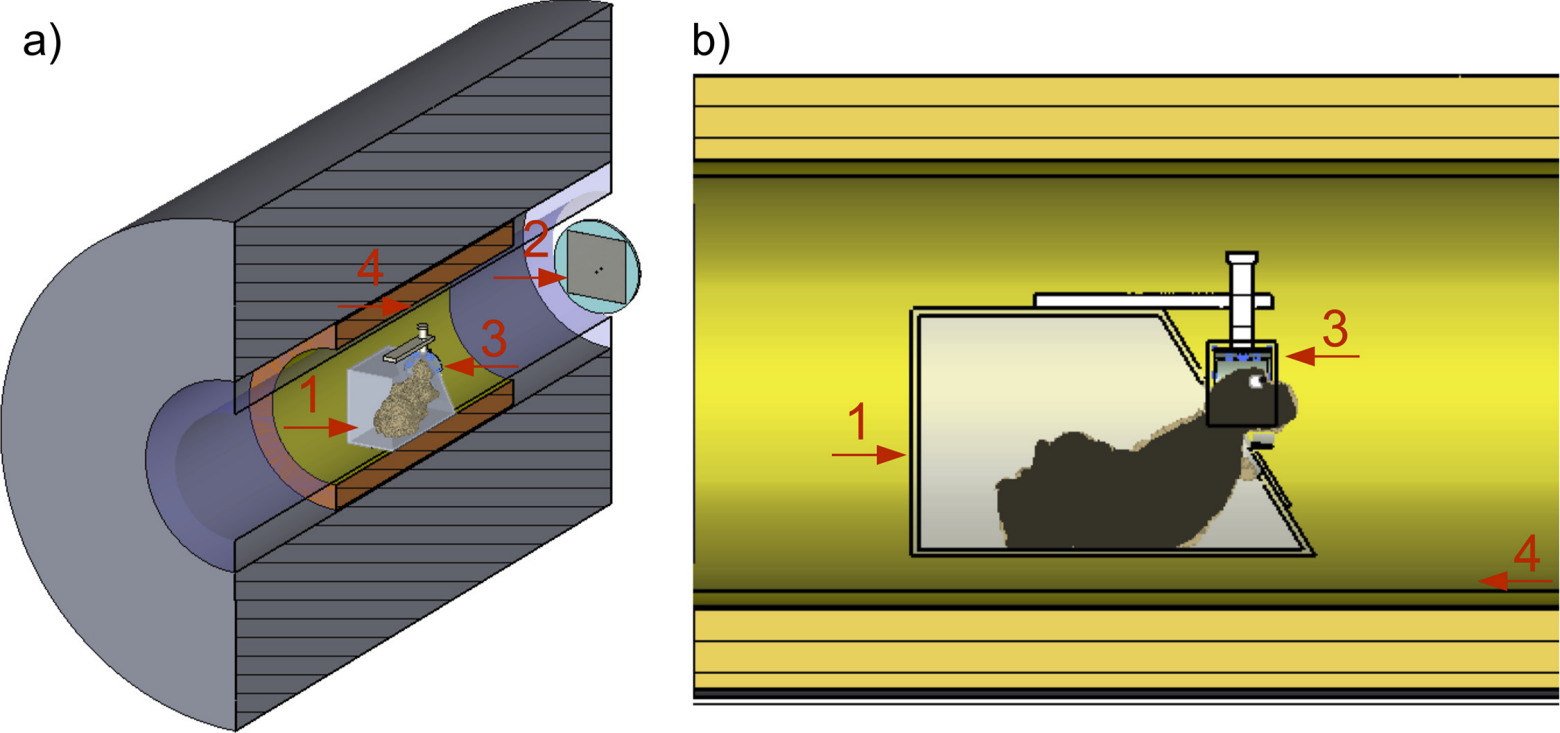
Simulation model for SAR validation of the TW Primate System in vivo setup. (**1**) Simulation model of a monkey. Body with extremities and the head were segmented from anatomic MRI data of a crab-eating macaque in a sphinx position with fixation device; Voxel were ascribed with the electromagnetic permittivity values for body (ε_r_ = 60) and eyes (ε_r_ = 69). (**2**) Patch antenna (**3**) 3-element phased array primate head coil; (**4**) RF shield of the gradient system. Perspective view (**a**). Side view (**b**).

In vivo experiments were performed on two adult female crab-eating macaques (Macaca fascicularis; monkeys, age 6–9 years, weight 5–7 kg). They were approved by the authority for animal care and ethics of the German federal state of Saxony-Anhalt (No. 28-42502-2-1129 IfN) and conformed to the rules for animal experimentation of the European Communities Council Directive (2010/63/EU). The monkeys were kept in a designated room of the animal house of the Leibniz Institute for Neurobiology. They were housed in compatible pairs or groups, with plenty of vertical space and perches for climbing and swinging and access to areas where they could play with toys or find privacy when resting. The cage was enriched with ropes, balls, boxes and sacks, some of which stuffed with dried fruit cubes, cereal (sugarless), popcorn, mealworms, seeds, grains, and nuts to allow the monkeys to forage for their food. Further enrichment was provided by a near cage located TV. The animals had unlimited access to water and food (fruits, vegetables, pellets). A veterinarian periodically (about every three months) examined the animals. During MRI measurements, which lasted 1.5 hours, monkeys were kept under general light anesthesia with a mixture of ketamine (2mg/kg) and xylazine (5mg/kg). Subsequently the monkeys woke from anesthesia and were returned to their home cage. The TW Primate System was used for one animal and the DH volume head coil for another. After induction of anesthesia, the monkey allocated to the TW Primate System was held in the sphinx position by using a fixation device and the monkey allocated to the monkey DH volume head coil in a side-lying position. Fig 5A shows the experimental setup for the TW Primate System on the 7 T MRI system. For imaging with both approaches, a B_1_^+^ flip angle mapping sequence based on a turbo FLASH sequence preceded by a magnetization preparation (Siemens work in progress package, provided by Hans-Peter Fautz) and a 3D turbo spin echo (TSE) sequence were used.

The turbo FLASH B_1_^+^ flip angle mapping sequence [16] was used with the following parameter settings: TR = 5000 ms, TE = 1.8 ms, matrix = 128x128, field of view (FoV) = 200x200 mm, slice thickness = 8 mm, α = 90°, rectangular pre-saturation pulse duration = 1 ms. The 3D TSE sequence was used with the following parameter settings: TE = 200 ms, TR = 4000 ms, matrix = 320x320, FoV = 150x150 mm, slice thickness = 0.5 mm, number of slices = 160, slice distance = 1 mm, voxel size: 0.47x0.47x0.5 mm, α = var. The 3D TSE sequence was used to obtain high SNR in an acceptable acquisition time (TA) while reducing the specific absorption rate (SAR) exposure [17]. To demonstrate the fMRI capability of the TW Primate System a 2D echo planar imaging (EPI) sequence [18] was used with the following parameter settings: TE = 23 ms, TR = 4500 ms, matrix = 200x200, FoV = 160x160 mm, slice thickness = 1 mm, number of slices = 30, slice distance = 0.2 mm, voxel size: 0.8x0.8x0.2 mm α = 90°, fat saturation = 40°.

### SAR evaluation

For in vivo UHF MRI measurements, the SAR is usually the most important limiting factor. For UHF human whole-body MRI systems, the legal limits are set to a maximum of 10 W/kg for local SAR of the head, averaged over 10 g of tissue [19]. The patch antenna generates an electromagnetic (EM) field inside the whole RF shield, which leads to an SAR-based limitation for the transmitted RF energy of the TW Primate System. Prior to the in vivo experiments, field simulation was used to calculate the SAR. The patch antenna (tuned and matched to the resonance frequency of 297.2 MHz) was designed using CST Microwave Studio 2014 software and the corresponding electromagnetic fields were calculated. To estimate the SAR for the in vivo experiments we used a biologically inspired monkey model with appropriate geometric and electromagnetic characteristics. This model was generated by a basic segmentation of a whole-body dataset acquired with an Achieva dStream 3 T (Philips, Best, Netherlands). The material parameters of the entire monkey model (head and body), except for the easily segmented eyes, were taken as averaged parameters to mimic the average properties of the monkey head and body based on human tissue material parameters [20] with ε_r_ = 60, ρ = 1040 kg/m^3^, σ = 0.8 S/m for head and body, and ε_r_ = 69, ρ = 1009 kg/m^3^, σ = 1.52 S/m for the eyes. This approach of simplifying the monkey tissue parameters to a few relevant tissue structures reduces the SAR prediction accuracy within an acceptable range [21]. Potential deviations of the SAR in the in vivo measurements were taken into account by adding a 20% safety range. For the TW Primate System the complete MRI system as described above was included in the simulation (Fig 5A). Fig 5B shows an axial cut through the monkey model, with the patch antenna on the right side and the monkey model with a fixation device at the bore center. With this setup we calculated the maximum operating performance of the patch antenna (accepted power, with minimum reflections to the excitation ports), while keeping the local SAR below the limits.

## Results

**Fig 6:**
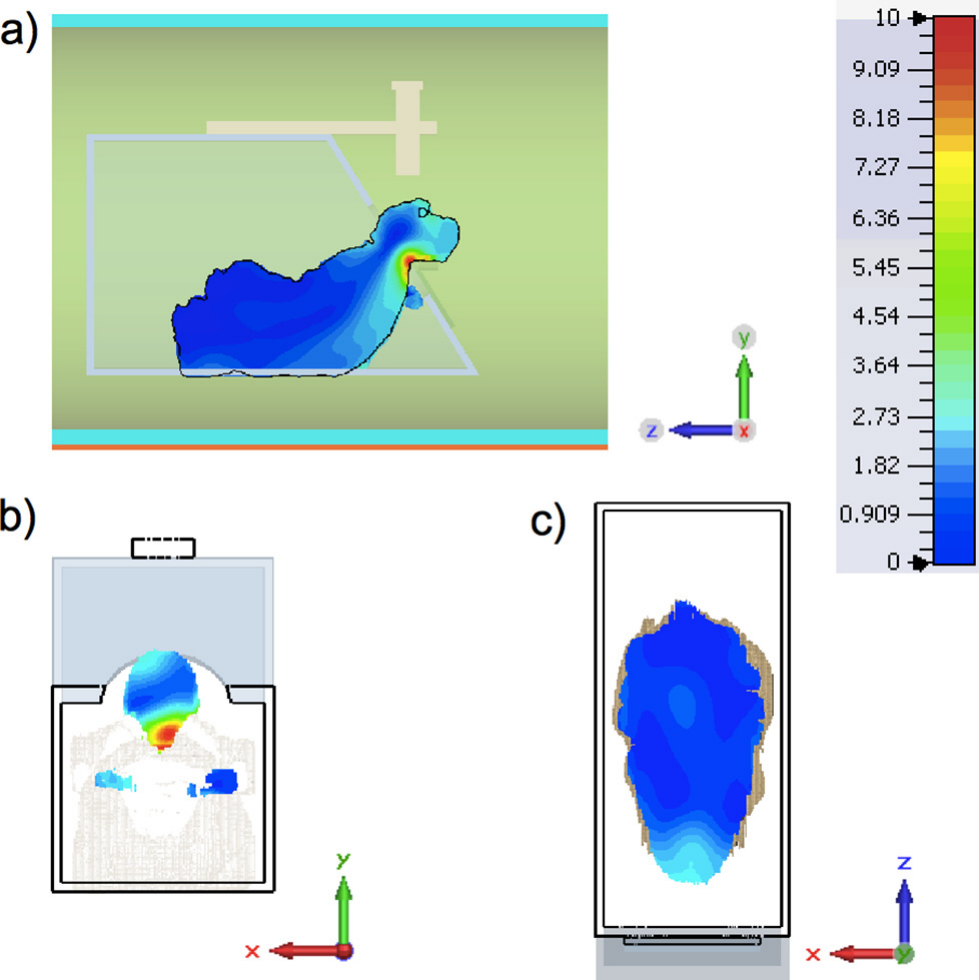
Color-encoded values of the SAR as simulated with the biological monkey model. Distribution of SAR on the sagittal plane cut in z-direction (**a**). Distribution of SAR on the head region transversal plane cut (**b**). A mid-coronal plane cut (**c**). For details of the simulation, see text.

Fig 6A presents the results of the SAR bioelectromagnetic (BioEM) field simulation showing the distribution of the SAR on a sagittal, centrally located slice through the biological monkey model. In Fig 6B and 6C the distribution of the maximum local SAR on a transversal and coronal are shown.

The SAR decreases strongly within an area of approx. 6 cm^2^ from the neck boundaries. A maximum value of 10 W/kg averaged over 10 g tissue can be detected on the head side, where the wave penetrates the biological monkey model first. To achieve the maximum of 10 W/kg local SAR, the patch antenna (matched to 50 Ω) has to radiate with an accepted RF power root mean square (RMS) of 249 W. Setting the excitation to the value of 249 W, the RF power absorbed by all lossy components inside the MRI system bore was 67 W. Only 7.8 W of the 67 W were absorbed by the simulated biological monkey model. The total whole-body SAR was 1.04 W/kg for the monkey model with a whole-body mass of 7.5 kg.

**Fig 7:**
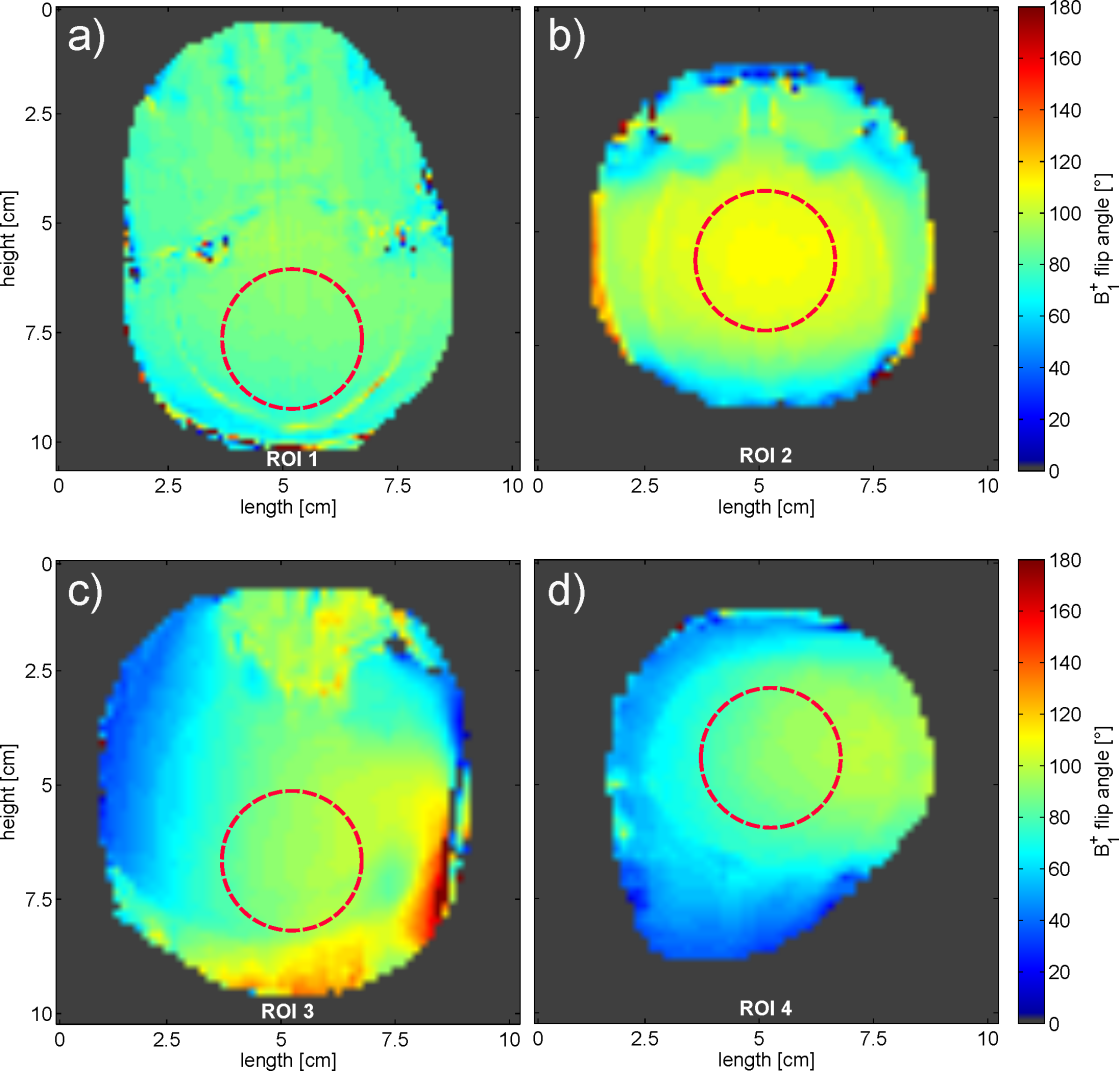
B1 flip angle map comparison of in vivo results from the TW Primate System and the Dual-Helmholtz primate volume head coil. Exemplary transversal slice of the B1 flip angle map in vivo results acquired with the TW Primate System (σ_α, ROI1_ = 3.1°) (**a**). Exemplary coronal slice of the B1 flip angle map (in vivo results acquired with the TW Primate System) (σ_α, ROI2_ = 3.9°) (**b**). Exemplary transversal slice of the B1 flip angle map acquired with the Dual-Helmholtz primate volume head coil (σ_α, ROI3_ = 5.8°) (**c**). Exemplary coronal slice of the B1 flip angle map acquired with the Dual-Helmholtz primate volume head coil (σ_α, ROI4_ = 5.9°) (**d**). (see S1 File)

The results of the in vivo B_1_ flip angle maps acquired with the TW Primate System and with the monkey volume head coil are shown in Fig 7A–7D, with two exemplary slices in the transversal and coronal plane of the crab-eating macaque brain. For a better comparison of the B_1_^+^ flip angle maps with the applied flip angle of 90°, the standard deviation σ_α_ was calculated for a circular ROI in the cerebrum with a 3 cm diameter. The standard deviations were σ_α_ = 3.1° in ROI1 (transversal plane) and σ_α_ = 3.9° in ROI2 (coronal plane) of the TW Primate System. The standard deviations of the monkey volume head coil were σ_α_ = 5.8° in ROI3 for the transversal plane and σ_α_ = 5.9° in ROI4 for the coronal plane.

**Fig 8:**
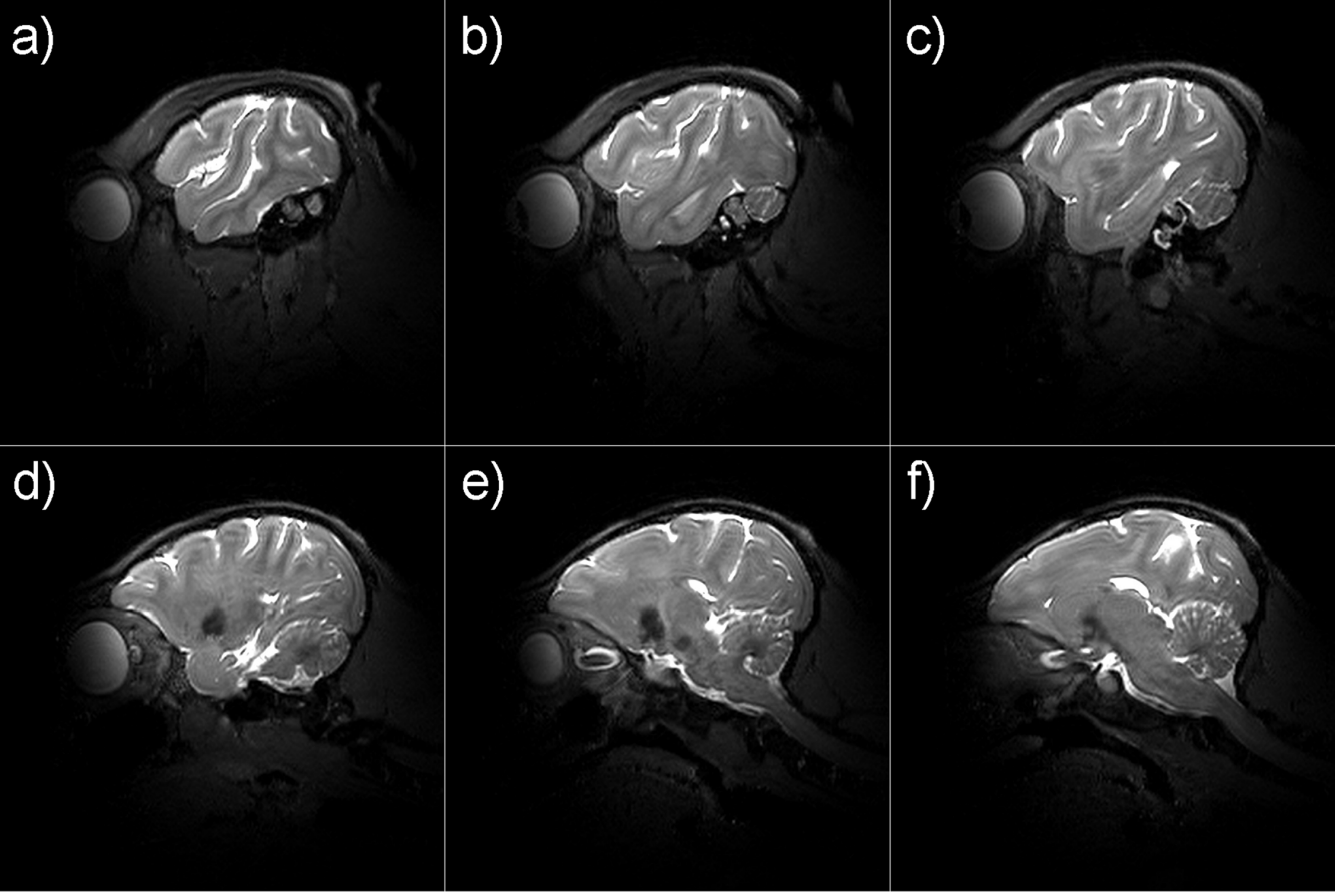
In vivo measurements using the TW Primate System. Exemplary slices of high-resolution 3D TSE of images of an anesthetized crab-eating macaque. Every tenth slice in the medial portion of the volume of interest (VOI) is shown to depict the main parts of the monkey brain. Gray and white matter, cerebellum and hippocampus are well delineated (**a-f**).

**Fig 9:**
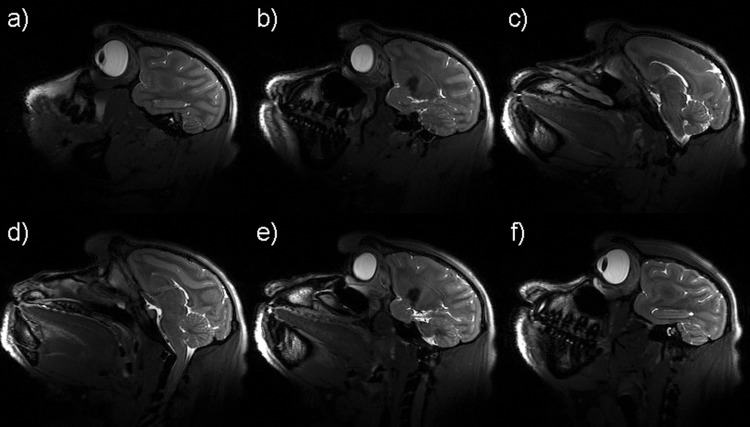
In vivo measurements using the Dual-Helmholtz primate volume head coil. Exemplary slices of high-resolution 3D TSE of images of an anesthetized crab-eating macaque Every tenth slice in the medial portion of the volume of interest (VOI) is shown to depict the main parts of the monkey brain. Gray and white matter, hippocampus, cerebellum and brain stem are well delineated (**a-f**).

**Fig 10:**
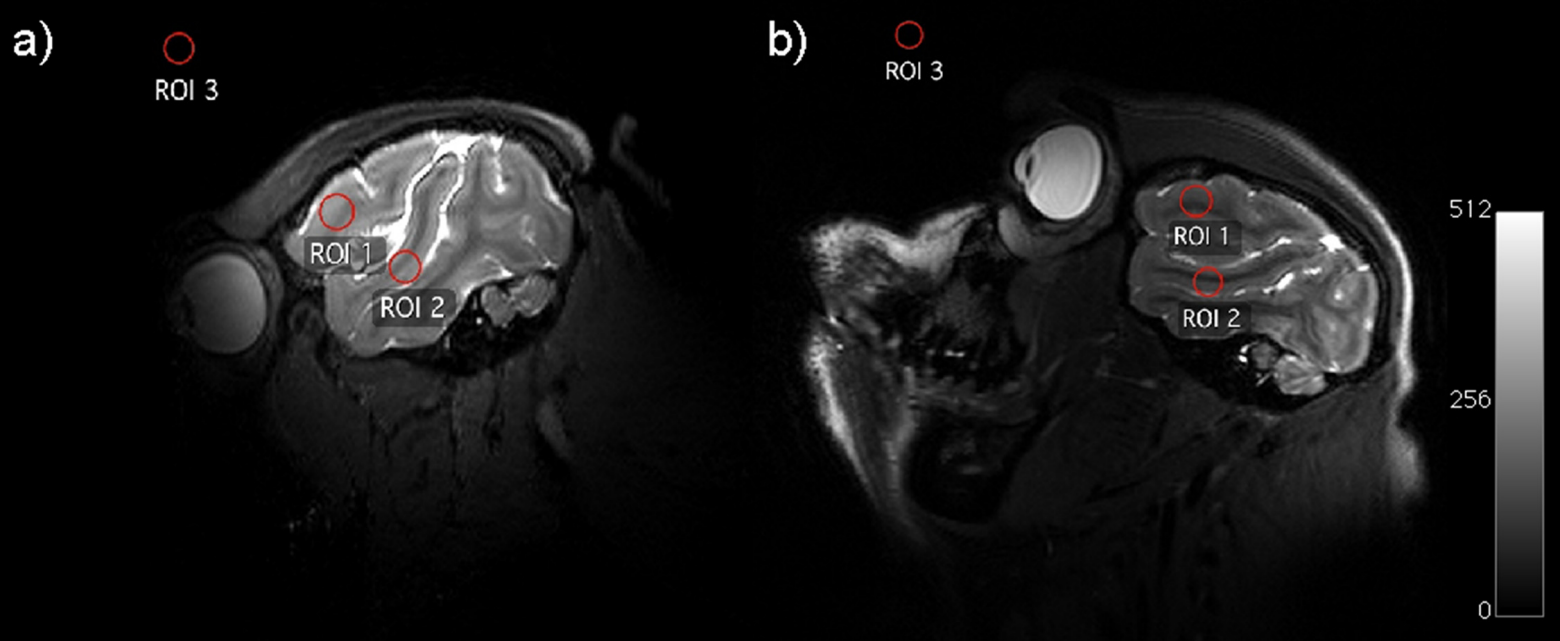
Comparison of the SNR in the high-resolution 3D TSE images from the TW Primate System and the Dual-Helmholtz primate volume head coil. Selected slice of the anatomical in vivo results of anesthetized crab-eating macaque for the TW Primate System (**a**). Selected slice for the Dual-Helmholtz primate volume head coil (**b**).

**Fig 11:**
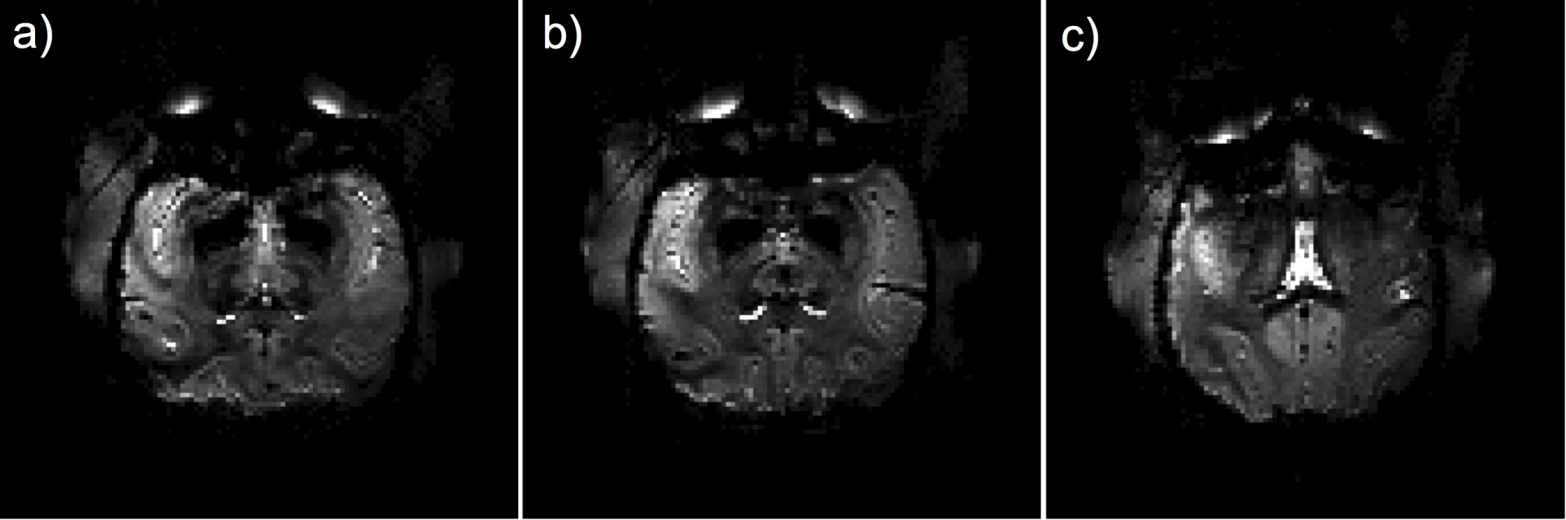
High-resolution 2D EPI in vivo images of an anesthetized crab-eating macaque acquired with the TW Primate System. Three exemplary consecutive coronal slices are displayed. Gray and white matter in the frontal and parietal lobes are well delineated. Images are not interpolated so that the original resolution is visible (**a-c**).

The results of the in vivo experiments with the TW Primate System and the 3D TSE sequence are shown in Fig 8A–8F, with six exemplary consecutive slices in sagittal plane of the macaque brain. The reference RMS voltage amplitude for the TW Primate System was U_ref_ = 270 V. Taking into account the calculated SAR limits, the available RF power of the system was sufficient to enable a 3D TSE method for anatomic imaging. For comparison, the results of the in vivo experiments with the monkey volume head coil and the 3D TSE sequence are shown in Fig 9A–9F, with six exemplary consecutive slices in sagittal plane of the macaque brain. The amplitude of the reference RMS voltage for the monkey volume head coil was U_ref_ = 197 V. For both approaches the images exhibit a high contrast and tissue homogeneity over the entire brain, depicting gray and white matter clearly. The low signal spot in Fig 8D and 8F and Fig 9B and 9E is localized in the striatum. The striatum has a lower signal intensity in T2-weighted images such as TSE (long TR and long TE). The high-resolution provides fine details of the anatomy. In addition to the sulci and gyri of the telencephalon, the cerebellum and the hippocampus are most apparent in the shown images; further smaller structures can be identified as well. The anatomical results of the monkey volume head coil also cover the brainstem and neck. For comparison of the sensitivity of the TW Primate System and monkey volume head coil, the SNR was measured in a selected 3D TSE image sum-of-squares combined dataset by the mean value approach [22]. Fig 10 shows two comparable slices, one acquired by the TW Primate System (Fig 10A), with μ_Pixel_ = 286 for ROI 1 (signal intensity gray matter), μ_Pixel_ = 262 for ROI 2 (signal intensity white matter), and μ_Pixel_ = 5.69 for ROI 3 (noise) and another acquired with the monkey volume head coil (Fig 10B), with μ_Pixel_ = 114 for ROI 1 (signal intensity gray matter), μ_Pixel_ = 126 for ROI 2 (signal intensity white matter), and μ_Pixel_ = 11.78 for ROI 3 (noise). For calculation of the SNR the associated background-noise coil-element correction factors for the mean value approach have to be applied [23]. This factor was CF_3E_ = 2.35 for the 3-element phased array coil and CF_12E_ = 4.85 for the 12-element phased array coil of the monkey volume head coil. The SNR of the TW Primate System in gray SNR_Gray_ = 118 and white matter SNR_White_ = 108 and that of the monkey volume head coil was SNR_Gray_ = 46 and SNR_White_ = 52. The results of the in vivo experiments for potential fMRI applications with the TW Primate System and the 2D EPI sequences are shown in Fig 11A–11C, with three exemplary consecutive slices in coronal plane of the crab-eating macaque brain. These images represent the entire brain of the crab-eating macaque, with a good depiction of gray and white matter mainly in the frontal and parietal lobes.

## Discussion

Our results show that the TW excitation approach [8] reliably facilitates excitation within a 7 T human whole-body UHF MRI system equipped with an SC72d whole-body gradient system. The patch antenna serves as a transmitter for a homogenous B_1_^+^ field in a central region of interest (ROI) with a diameter of about 10 cm. The B_1_^+^ field homogeneity in this central ROI is nearly equivalent to the "gold-standard" birdcage architecture, as shown in Mallow et al. 2013 [9].

This good B_1_^+^ field homogeneity was used for rodent imaging at 16.4 T with a 26 cm horizontal bore magnet attached to a BioSpec spectrometer (Bruker BioSpin MRI GmbH, Germany) in Shajan et al. 2012 [24]. In addition, the TW approach was used on a 9.4 T (400 MHz) whole-body MRI system (Siemens Healthcare, Erlangen, Germany) that was equipped with the whole-body gradient system introduced by Geschewski et al. 2013 [25] and on a similar whole-body MRI system equipped with an AC84 head-only gradient system introduced by Hoffmann et al. 2012 [26]. Hoffmann et al. [26] implemented the TW approach for human head imaging with the AC84 head-only gradient insert with a 40 cm diameter RF shield. Unlike to a whole-body gradient system, RF shield diameter was thus decreased, leading to an increase of the cutoff frequency to 440 MHz and therefore to an adverse reduction of RF performance. The conditions on a 7 T whole-body MRI system are well suited for animal imaging when neuroimaging of macaques is the primary interest. However, some prerequisites have to be fulfilled for this approach. The TW Primate System works only in those UHF MRI systems that match the geometric conditions for the propagation of the circular TE_11_ waveguide mode [9]. This means that, depending on the excitation frequency, the diameter of the gradient RF shield needs to be larger than 60 cm diameter, which is not the case in all human 7 T UHF MRI systems. Due to increased SAR and the inhomogeneous field distribution under UHF conditions, no commercial UHF whole-body MRI system possesses a body coil resonator. Vaughan et al. 2009 [27] showed that using the transverse electromagnetic (TEM) volume coil design as a body coil in a UHF MRI system resulted in a good B_1_^+^ transmission efficiency. Compared with the TW excitation approach, however, the TEM design has fewer degrees of freedom because its architecture requires more space inside the inner bore of the MRI system. Furthermore, TEM is more expensive than the TW excitation approach. Similarly, Orzada et al. 2013 [28] demonstrated the usability of an eight-channel transmit coil array in combination with B_1_-shimming for human body imaging. Compared with the TW excitation approach, this setup delivers good results for human body imaging but would not be suitable for monkey imaging in combination with a fixation device. The search for a fully sufficient UHF body coil resonator is thus an ongoing process.

Instead, RF coils consisting of a transmit volume coil in combination with phased array receive coil are the standard architecture to provide homogeneous excitation and the receiving sensitivity required for imaging in UHF conditions. However, these coils usually enclose the object tightly (to achieve highest sensitivity), leaving less space for additional experimental equipment as needed for auditory and visual brain research. By contrast, more space is available in the TW Primate System, in which it is also possible to install additional devices. Other appealing advantages include the possibility to use a fixation device for fMRI in the sphinx position as well as the much lower costs for the components and the production of a patch antenna compared with those for a volume head coil in birdcage-like architecture. The sphinx position for imaging of the human head with the TW excitation approach was already presented by Zhang et al. [29] but is seldom used because this position is not comfortable for humans in studies entailing extended periods of time. As a further advantage, the patch antenna in combination with the appropriate high sensitive phased array coil is usable in every experimental setup.

The results of the B_1_ flip angle maps show a sufficient homogeneity of the applied flip angle of 90° in the central brain region for both excitation approaches. The TW Primate System showed a slightly better homogeneity in comparison to the monkey volume head coil. This difference is partly due to the non-optimal monkey head position inside the monkey volume head coil.

A comparison of the in vivo B_1_^+^ transmission efficiency of both imaging approaches shows a 37% higher B_1_^+^ transmission efficiency for the monkey volume head coil, with B_1_PVHC_ = 13.3 μT/√kW (U_ref_ = 197 V) for it and B_1_TWPS_ = 9.7 μT/√ kW (U_ref_ = 270 V) for the TW Primate System. The B_1_^+^ transmission efficiency is given in [μT/√kW] and can be calculated by the measured reference voltage (U_ref_) that represents the needed RF energy for a 180° pulse (B_1_ = 11.75 μT) converted in μT divided by the square root RMS power √P in kW. The lower B_1_^+^ transmission efficiency of the TW excitation is due to a smaller B_1_^+^ filling factor for the volume-of-interest. In comparison with volume resonators, the according B_1_^+^ transmission efficiency decreases—a finding also reported by other groups [30],[31]—which constitutes the main disadvantage of the TW excitation approach. The 37% B_1_^+^ transmission efficiency gap between both imaging approaches increases dramatically to approx. 93% by using low dielectric materials such as silicon oil, as shown in Mallow et al. 2013 [9]. This leads to the conclusion that the TW excitation approach is effective only in combination with high dielectric materials such as water containing tissue. To avoid B_1_^+^ homogeneities in high dielectric tissues, the degrees of freedom for B_1_^+^-shimming are limited by the provided RF power and low B_1_^+^ transmission efficiency [32]. Recently, interesting alternative approaches were proposed to increase the latter's B_1_^+^ transmission efficiency [33]. Furthermore, the SAR results show the same low RF efficiency of the TW excitation approach in comparison to the more efficient volume resonator structures. Only a small amount of the RF energy radiated by the patch antenna is dissipated in the lossy tissue material of the monkey body. The RF energy balance is much better for resonator coils than for the TW excitation approach. The T2-weighed in vivo 3D TSE sequence measurements showed that high-resolution anatomic images could be acquired for both coil architectures: The TW Primate System and the monkey volume head coil provided high contrast and tissue homogeneity over the entire brain for a clear depiction of gray and white matter. The high-resolution provides fine details of the anatomy. Besides the sulci and gyri of the telencephalon, the cerebellum and the hippocampus are clearly visible in the images. Given its larger array size, the monkey volume head coil covers the brainstem and neck. This coverage is dominated by the receive coil. This slightly bigger relative field of view (FoV) may be an advantage if those regions have to be examined. However, most monkey MRI focuses on functional imaging and is thus mainly interested in the cerebrum. In the gray matter, the SNR_Gray_ = 118 of the TW Primate System is approx. three times higher than that of the monkey volume head coil with SNR_Gray_ = 47. In the white matter, the SNR is approx. twice as high for the TW Primate System with SNR_White_ = 108 and SNR_White_ = 52 for the monkey volume head coil. One reason for this large difference in sensitivity is the non-optimal monkey head position inside the monkey volume head coil and the larger inner diameter of the receive coil consisting of 12 elements. Even the coil correction factors cannot compensate these experimental boundary conditions. These correction factors eliminate the additionally added background noise from the sum-of-square combined element signals if the coil fits optimally to the load; using a coil with more elements normally increases the SNR. The monkey volume head coil allows a limited degree of positioning of the macaque in return for an optimum imaging position of the anaesthetized monkey. Its major disadvantage is that there is no option for using a sphinx position. The 2D EPI in vivo measurements were acquired to test whether functional imaging as the major neuroimaging application would be feasible with the TW approach. The images show that most parts of the brain are well mapped, even showing a delineation between gray and white matter mainly in the frontal and parietal lobes. The caudal parts exhibit increasing B_0_-shiming artifacts, but these may be reduced by optimizing the image sequence parameter and B_0_-shim.

Motion artifacts that affect the B_0_ field could also decrease the image quality for awake monkey fMRI. The fixation device in combination with the sphinx position reduces normal motion artifacts and artifacts from breathing, but not those from jaw motion. The latter artifacts can be reduced by intensive conditioning of the monkey and a jaw motion sensor in combination with image motion correction technics, as shown by Keliris et al. 2007 [14,34,35]. This issue could be more relevant when using the horizontal MRI system with a sphinx position in comparison to vertical MRI systems with the monkey in a more natural sitting position. Motion artifacts remain a challenge for both the TW Primate System and the conventional volume coil approach, which are equally susceptible to them. Monkey conditioning and application of a jaw motion sensor in combination with image motion correction techniques can improve the results of the TW Primate System in the future. However, our limited experimental time budget did not allow us to training monkeys or develop and validated such a jaw motion sensor in combination with image motion correction in order to reduce motion artifacts, so we were not yet able to run in vivo fMRI with awake monkeys.

The experimental procedure also emphasized one of the major advantages of the TW Primate System: the easy positioning of the monkey inside the MRI system because the sphinx position is not disturbed when using the 3-element phased array receive-only head coil. In comparison with a conventional volume head coil concept, the 3-element phased array head coil leaves more space for additional equipment and resulted in a higher SNR. The SNR in gray and white matter (SNR_Gray_ = 118, SNR_White_ = 108) acquired with the TW Primate System was higher than the SNR of Kolster et al. 2009 [12], namely 35 and 80 for a 4- and 8-element receive coil inside the cerebrum. Kolster et al. 2009 used a 7 T whole-body MRI in combination with a surface coil for Tx and a 4- and 8-element receive coil for fMRI of rhesus monkeys in the sphinx position. The difference in SNR is mainly attributable to the different excitation methods. A surface coil as heterogeneous resonator for Tx, as used in the study of Kolster et al. 2009, cannot generate the same flip angle across the whole monkey brain.

As shown by Pfeuffer et al. 2004, the achieved SNR and image quality can be increased by using implanted coils and a better gradient system [36]. This high SNR is caused by the high receive B_1_ filling factor provided by the monkey head being tightly fixated as well as the fact that the preamplifier could be installed in close proximity to the RF coil. The use of this special 3-element phased array head coil also leads to a higher flexibility in presenting stimuli in functional MRI, e.g. when using earphones or visual stimulation devices. In comparison to vertical magnet MRI systems [3], horizontal UHF magnet MRI systems with the presented TW Primate System may provide an improvement if more space is needed for stimulation devices or when scanning larger monkeys (or even two norm-sized monkeys in a same scan).

The results of this work thus add additional evidence that for examination of non-human primates a human whole-body MRI system may represent a good alternative, especially as the equipment is cost-effective and 7 T UHF scanners are becoming much more widely available. Vertical monkey MRI systems with smaller gradient system such as the 7 T vertical MRI with a 38 cm inner diameter (Bruker BGA-38) provide better imaging conditions in terms of the gradient system and B_0_-shim quality, which directly affects the EPI quality (T2* imaging). In contrast, achieving a high B_0_ homogeneity is more difficult when placing the relatively small monkey inside the larger bore of the human 7 T whole-body MRI system. However, B_0_-shimming up to the 3rd order may still facilitate increasing the field homogeneity on a 7 T whole-body MRI system with a 60 cm diameter, as described by Sengupta et al. 2011 and Pan et al. 2012 [37,38].

The vertical gradient system used by Pfeuffer et al. 2004 [36] provides 80 mT/m with a slew rate of 400 mT/m/ms, which increases image quality considerably compared to our horizontal MRI gradient system with a maximum amplitude of 70 mT/m and maximum slew rate of 200 mT/m/ms. This difference is significant for SNR and EPI quality. The optimized vertical MRI systems are dedicated exclusively to specialized animal experiments. A further optimization of the TW Primate System should render the presented approach a promising new alternative for research groups aspiring to perform monkey MRI but having access solely to horizontal human whole-body UHF MRI systems.

## Supporting Information

S1 File B1 flip angle maps (DICOM format) of the TW Primate System and the Dual-Helmholtz primate volume head coil. The transversal and coronal slices. (TW_Tra.dcm; TW_Cor.dcm; DH_Tra.dcm; DH_Cor.dcm).
(ZIP)

S2 File Fig 1 and Fig 7 as EPS vector format file. (ZIP)

## References

[1] Ogawa S, Lee TM, Kay AR, Tank DW. Brain magnetic resonance imaging with contrast dependent on blood oxygenation. Proc Natl Acad Sci U S A. 1990;87: 9868–9872. 2124706 10.1073/pnas.87.24.9868 PMC55275

[2] Goense JBM, Ku S-P, Merkle H, Tolias AS, Logothetis NK. fMRI of the temporal lobe of the awake monkey at 7 T. NeuroImage. 2008;39: 1081–1093. 10.1016/j.neuroimage.2007.09.038 18024083

[3] Kagan I, Iyer A, Lindner A, Andersen RA. Space representation for eye movements is more contralateral in monkeys than in humans. Proc Natl Acad Sci. 2010;107: 7933–7938. 10.1073/pnas.1002825107 20385808 PMC2867911

[4] Vanduffel W, Fize D, Mandeville JB, Nelissen K, Van Hecke P, Rosen BR, et al Visual Motion Processing Investigated Using Contrast Agent-Enhanced fMRI in Awake Behaving Monkeys. Neuron. 2001;32: 565–577. 10.1016/S0896-6273(01)00502-5 11719199

[5] Hayes CE. The development of the birdcage resonator: a historical perspective. NMR Biomed. 2009;22: 908–918. 10.1002/nbm.1431 19856386

[6] Roemer PB, Edelstein WA, Hayes CE, Souza SP, Mueller OM. The NMR phased array. Magn Reson Med. 1990;16: 192–225. 10.1002/mrm.1910160203 2266841

[7] Orzada S, Maderwald S, Göricke SL, Parohl N, Ladd SC, Ladd ME, et al Design and comparison of two eight-channel transmit/receive radiofrequency arrays for in vivo rodent imaging on a 7 T human whole-body MRI system. Med Phys. 2010;37: 2225–2232. 20527556 10.1118/1.3378478

[8] Brunner DO, Zanche ND, Fröhlich J, Paska J, Pruessmann KP. Travelling-wave nuclear magnetic resonance. Nature. 2009;457: 994–998. 10.1038/nature07752 19225521

[9] Mallow J, Herrmann T, Kim K-N, Stadler J, Mylius J, Brosch M, et al Ultra-high field MRI for primate imaging using the travelling-wave concept. Magn Reson Mater Phys Biol Med. 2013;26: 389–400. 10.1007/s10334-012-0358-z 23233135

[10] Purcell EM, Torrey HC, Pound RV. Resonance Absorption by Nuclear Magnetic Moments in a Solid. Phys Rev. 1946;69: 37–38. 10.1103/PhysRev.69.37

[11] CST AG. CST Microwave Studio 2014. 2014;

[12] Kolster H, Mandeville JB, Arsenault JT, Ekstrom LB, Wald LL, Vanduffel W. Visual Field Map Clusters in Macaque Extrastriate Visual Cortex. J Neurosci Off J Soc Neurosci. 2009;29: 7031–7039. 10.1523/JNEUROSCI.0518-09.2009 PMC2749229 19474330

[13] Denys K, Vanduffel W, Fize D, Nelissen K, Peuskens H, Essen DV, et al The Processing of Visual Shape in the Cerebral Cortex of Human and Nonhuman Primates: A Functional Magnetic Resonance Imaging Study. J Neurosci. 2004;24: 2551–2565. 10.1523/JNEUROSCI.3569-03.2004 15014131 PMC6729498

[14] Keliris GA, Shmuel A, Ku S-P, Pfeuffer J, Oeltermann A, Steudel T, et al Robust controlled functional MRI in alert monkeys at high magnetic field: Effects of jaw and body movements. NeuroImage. 2007;36: 550–570. 10.1016/j.neuroimage.2007.02.057 17509896

[15] Cho Z-H, Han J-Y, Hwang S-I, Kim D, Kim K-N, Kim N-B, et al Quantitative analysis of the hippocampus using images obtained from 7.0 T MRI. NeuroImage. 2010;49: 2134–2140. 10.1016/j.neuroimage.2009.11.002 19909820

[16] Klose U. Mapping of the radio frequency magnetic field with a MR snapshot FLASH technique. Med Phys. 1992;19: 1099–1104. 10.1118/1.596828 1518473

[17] Hennig J. Multiecho imaging sequences with low refocusing flip angles. J Magn Reson 1969. 1988;78: 397–407. 10.1016/0022-2364(88)90128-X

[18] Mansfield P. Real-time echo-planar imaging by NMR. Br Med Bull. 1984;40: 187–190. 6744006 10.1093/oxfordjournals.bmb.a071970

[19] IEC. Medical electrical equipment—part 2–33 particular requirements for the safety of magnetic resonance equipment for medical diagnosis. 3rd ed. 2010.

[20] Gabriel S, Lau RW, Gabriel C. The dielectric properties of biological tissues: II. Measurements in the frequency range 10 Hz to 20 GHz. Phys Med Biol. 1996;41: 2251 10.1088/0031-9155/41/11/002 8938025

[21] Wolf S, Diehl D, Gebhardt M, Mallow J, Speck O. SAR simulations for high-field MRI: How much detail, effort, and accuracy is needed? Magn Reson Med. 2013;69: 1157–1168. 10.1002/mrm.24329 22611018

[22] Constantinides CD, Atalar E, McVeigh ER. Signal-to-Noise Measurements in Magnitude Images from NMR Phased Arrays. Magn Reson Med Off J Soc Magn Reson Med Soc Magn Reson Med. 1997;38: 852–857. 10.1002/mrm.1910380524 PMC2570034 9358462

[23] Dietrich O, Raya JG, Reeder SB, Ingrisch M, Reiser MF, Schoenberg SO. Influence of multichannel combination, parallel imaging and other reconstruction techniques on MRI noise characteristics. Magn Reson Imaging. 2008;26: 754–762. 10.1016/j.mri.2008.02.001 18440746

[24] Shajan G, Hoffmann J, Balla DZ, Deelchand DK, Scheffler K, Pohmann R. Rat brain MRI at 16.4T using a capacitively tunable patch antenna in combination with a receive array. NMR Biomed. 2012;25: 1170–1176. 10.1002/nbm.2786 22344898

[25] Geschewski FH, Brenner D, Felder J, Jon Shah N. Optimum coupling and multimode excitation of traveling-waves in a whole-body 9.4T scanner. Magn Reson Med. 2013;69: 1805–1812. 10.1002/mrm.24403 22782491

[26] Hoffmann J, Shajan G, Budde J, Scheffler K, Pohmann R. Human brain imaging at 9.4 T using a tunable patch antenna for transmission. Magn Reson Med. 2012; n/a–n/a. 10.1002/mrm.24367 22706783

[27] Vaughan JT, Snyder CJ, DelaBarre LJ, Bolan PJ, Tian J, Bolinger L, et al 7 T Whole Body Imaging: Preliminary Results. Magn Reson Med Off J Soc Magn Reson Med Soc Magn Reson Med. 2009;61: 244–248. 10.1002/mrm.21751 PMC2875945 19097214

[28] Orzada S, Johst S, Maderwald S, Bitz AK, Solbach K, Ladd ME. Mitigation of B1+ inhomogeneity on single-channel transmit systems with TIAMO. Magn Reson Med. 2013;70: 290–294. 10.1002/mrm.24453 22886695

[29] Zhang B, Brown R, Wiggins C, Sodickson DK, Stoeckel B, Wiggins GC. A 7T Coil System for Imaging Humans in the Sphinx Position to Evaluate the Effect of Head Orientation Relative to B0 for MR Imaging. 19th Annu ISMRM Sci Meet Exhib 2011 Vol 3. 2011; 162.

[30] Alt S, Müller M, Umathum R, Bolz A, Bachert P, Semmler W, et al Coaxial waveguide MRI. Magn Reson Med. 2012;67: 1173–1182. 10.1002/mrm.23069 22021117

[31] Brunner DO, Paška J, Froehlich J, Pruessmann KP. Traveling-wave RF shimming and parallel MRI. Magn Reson Med. 2011;66: 290–300. 10.1002/mrm.22817 21695729

[32] Orzada S, Maderwald S, Poser BA, Bitz AK, Quick HH, Ladd ME. RF excitation using time interleaved acquisition of modes (TIAMO) to address B1 inhomogeneity in high-field MRI. Magn Reson Med. 2010;64: 327–333. 10.1002/mrm.22527 20574991

[33] Erni D, Liebig T, Rennings A, Koster NHL, Frohlich J. Highly adaptive RF excitation scheme based on conformal resonant CRLH metamaterial ring antennas for 7-Tesla traveling-wave magnetic resonance imaging. 2011 Annual International Conference of the IEEE Engineering in Medicine and Biology Society,EMBC. 2011. pp. 554–558. 10.1109/IEMBS.2011.6090102 22254370

[34] Schulz J, Siegert T, Bazin P-L, Maclaren J, Herbst M, Zaitsev M, et al Prospective slice-by-slice motion correction reduces false positive activations in fMRI with task-correlated motion. NeuroImage. 2014;84: 124–132. 10.1016/j.neuroimage.2013.08.006 23954484

[35] Maclaren J, Herbst M, Speck O, Zaitsev M. Prospective motion correction in brain imaging: A review. Magn Reson Med. 2013;69: 621–636. 10.1002/mrm.24314 22570274

[36] Pfeuffer J, Merkle H, Beyerlein M, Steudel T, Logothetis NK. Anatomical and functional MR imaging in the macaque monkey using a vertical large-bore 7 Tesla setup. Magn Reson Imaging. 2004;22: 1343–1359. 10.1016/j.mri.2004.10.004 15707785

[37] Pan JW, Lo K-M, Hetherington HP. Role of very high order and degree B0 shimming for spectroscopic imaging of the human brain at 7 tesla. Magn Reson Med. 2012;68: 1007–1017. 10.1002/mrm.24122 22213108 PMC3323711

[38] Sengupta S, Avison MJ, Gore JC, Brian Welch E. Software compensation of Eddy current fields in multislice high order dynamic shimming. J Magn Reson. 2011;210: 218–227. 10.1016/j.jmr.2011.03.007 21458339 PMC3098125

